# Effect of Lu-Doping on Electrical Properties of Strontium Zirconate

**DOI:** 10.3390/membranes13070663

**Published:** 2023-07-12

**Authors:** Anastasiya Pavlovich, Alexander Pankratov, Liliya Dunyushkina

**Affiliations:** Institute of High Temperature Electrochemistry, Ural Branch of the Russian Academy of Sciences, 20 Akademicheskaya St., 620066 Ekaterinburg, Russia; nastenka_98@mail.ru (A.P.); a.pankratov@ihte.uran.ru (A.P.)

**Keywords:** Lu-doped SrZrO_3_, proton-conducting membrane, perovskite, proton conductivity, ionic conductivity

## Abstract

SrZrO_3_-based perovskites are promising proton-conducting membranes for use in fuel and electrolysis cells, sensors, hydrogen separators, etc., because they combine good proton conductivity with excellent chemical stability. In the present research, the effect of Lu-doping on microstructure, phase composition, and electrical conductivity of SrZr_1−x_LuxO_3−δ_ (x = 0–0.10) was investigated via X-ray diffraction, scanning electron microscopy, energy-dispersive X-ray spectroscopy and impedance spectroscopy. Dense ceramic samples were obtained by the solution combustion synthesis and possessed an orthorhombic perovskite-type structure. The solubility limit of Lu was revealed to lie between x = 0.03 and 0.05. The conductivity of SrZr_1−x_Lu_x_O_3−δ_ increases strongly with the addition of Lu at x < 0.05 and just slightly changes at x > 0.05. The rise of the water vapor partial pressure results in an increase in the conductivity of SrZr_1−x_LuxO_3−δ_ ceramics, which confirms their hydration ability and significant contribution of protonic defects to the charge transfer. The highest conductivity was achieved at x = 0.10 (10 mS cm^–1^ at 700 °C, wet air, pH_2_O = 0.61 kPa). The conductivity behavior was discussed in terms of the defect formation model, taking into account the improvement in ceramic sintering at high lutetium concentrations.

## 1. Introduction

Direct conversion of chemical energy into electricity by using proton-conducting solid oxide fuel cells (PC-SOFCs) is an efficient and environmentally friendly technology [1,2,3]. When hydrogen which is a zero-carbon fuel is consumed in a fuel cell, only water is produced; therefore, the use of the hydrogen-fueled PC-SOFCs for energy production allows for reducing greenhouse gas emissions. So far as hydrogen is an ideal fuel, efficient technologies of hydrogen production are required. Water electrolysis by using proton-conducting solid oxide electrolysis cells (PC-SOECs) is a promising way to convert and store energy [4,5,6]. A solid electrolyte with high proton conductivity and excellent chemical stability is required for the reliable and long-term operation of PC-SOFCs/SOECs. Apart from the fuel and electrolysis cells, proton-conducting membranes can be used in hydrogen separators [7], hydrogen sensors [8], hydrogen pumps [9], etc.

SrZrO_3_-based perovskites are recognized as promising membranes for proton-conducting electrochemical devices because they combine appreciable proton conductivity with excellent chemical stability [10,11,12,13,14,15,16,17,18,19]. Oxide-ion conductivity in solid oxides is realized due to the presence of oxygen vacancies in the structure. In the undoped zirconates of alkaline earth elements, the oxide-ion conductivity is low because of a small concentration of oxygen vacancies. Acceptor doping on the B-sites in the perovskite-type A^2+^B^4+^O_3_ oxides results in the appearance of oxygen vacancies as follows:

(1) $$M_{2}O_{3}\to 2M_{\mathrm{Zr}}^{/}+V_{O}^{••},$$

where $M_{\mathrm{Zr}}^{/}$ is a substitution defect and $V_{O}^{••}$ denotes an oxygen vacancy.

Protonic defects in perovskites are generated in water vapor-containing atmosphere due to the hydration reaction:

(2) $$H_{2}O\left(g\right)+V_{O}^{••}+O_{O}^{\times }=2{\mathrm{OH}}_{O}^{•},$$

where ${\mathrm{OH}}_{O}^{•}$ denotes a positively charged protonic defect and $O_{O}^{\times }$ is a lattice oxygen ion.

According to the law of mass action for reactions (1) and (2), the concentration of protonic defects increases with the concentration of oxygen vacancies, and therefore with the dopant concentration, and with an increase in the water vapor partial pressure (pH_2_O). That is why acceptor doping is commonly used to improve the proton conductivity in the perovskite-type oxides. In recent years, the replacement of zirconium with trivalent metals in SrZrO_3_ has been extensively investigated due to the high potential for application in electrochemical devices. For example, doping with Y was shown to significantly improve the ionic conductivity of strontium zirconates [11,13,14]. SrZrO_3_ doped with 5 mol.% Gd demonstrated an appreciable proton conductivity [16]. Substitution of Zr for Yb and Dy also resulted in an increase in the conductivity of SrZrO_3_ [17,18]. Doping with 5 mol.% Yb and Y was revealed to result in a larger gain in conductivity compared to the same concentrations of In, Al and Ga [12]. It is worth emphasizing that although SrZrO_3_-based perovskites are inferior in conductivity to strontium cerates, zirconates are more resistant to the formation of carbonates and hydroxides in the presence of CO_2_ and H_2_O in the atmosphere [1], and have higher ion transference numbers [12,13], which are serious advantages for potential applications as a proton-conducting membrane.

Although the positive effect of doping SrZrO_3_ with various rare earth elements on electrical conductivity has been studied, doping with lutetium has not yet been considered. That is why the aim of the present research is to examine the effect of Lu-doping on the charge transport properties of strontium zirconate. For this, the ceramic samples of SrZr_1−x_Lu_x_O_3−δ_ (x = 0, 0.03, 0.05, 0.07, and 0.10) were fabricated by the solution combustion synthesis and sintering at 1650 °C, and their phase purity, crystal structure, microstructure, chemical composition, and electrical conductivity were studied by using X-ray diffraction (XRD), scanning electron microscopy (SEM), energy dispersive X-ray spectroscopy (EDX), and impedance spectroscopy methods. The conductivity behavior of SrZr_1−x_Lu_x_O_3−δ_ was discussed and compared to other proton-conducting electrolytes.

## 2. Experimental

**Figure 1:**
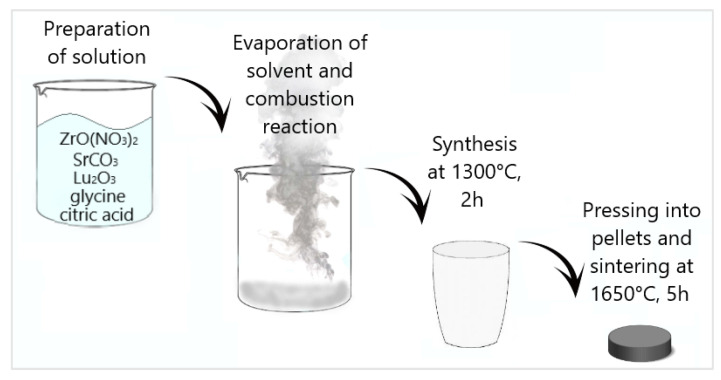
Schematic diagram of SrZr_1−x_Lu_x_O_3−δ_ synthesis.

SrZr_1−x_Lu_x_O_3−δ_ (x = 0, 0.03, 0.05, 0.07, and 0.10) powders were synthesized through the solution combustion synthesis using SrCO_3_, ZrO(NO_3_)_2_∙xH_2_O and Lu_2_O_3_ (all with 99.9% purity) as precursors. Glycine (99% purity) and citric acid (99% purity) were used as a fuel and a complexant, respectively. The stoichiometric composition of SrCO_3_ and Lu_2_O_3_ powders was added to the aqueous solution of zirconyl nitrate. The mixture was slowly heated until complete dissolution, and then glycine and citric acid were added. The molar ratio of metal ions, glycine and citric acid was 2:2:1. To evaporate the solvent, the solution was held on a heating plate at 90 °C and periodically stirred. The obtained viscous gel was slowly heated until a self-propagating combustion reaction was initiated. The resulting powders were synthesized at 1300 °C for 2 h. A schematic diagram of the synthesis is presented in Figure 1. After synthesis, the samples were thoroughly ground in an agate mortar, uniaxially pressed into pellets under a pressure of 300 MPa and sintered at a temperature of 1650 °C for 5 h in ambient air.

In order to calculate the apparent density of the sintered samples, their mass, thickness and diameter were measured. The relative density was calculated as the ratio of an apparent density to a theoretical one. 

The phase purity of the synthesized powders was studied by using X-ray diffraction carried out on a diffractometer D-Max 2200 (Rigaku, Tokyo, Japan) in Cu-kα radiation at a scanning speed of 0.02 rpm with a step of 0.1° over 2θ range from 10 to 90°. 

Microstructural study was carried out using scanning electron microscopy (SEM) on a MIRA 3 LMU (Tescan, Brno, Czech Republic). For SEM analysis, the sintered ceramics were first polished with a diamond paste and then calcined at a temperature of 1300 °C for 2 h for making grains well visible. The chemical composition of the sintered samples was calculated from EDX data, which were measured and averaged for 10 spots. 

Electrical properties of the samples were characterized by using the impedance spectroscopy method. For the electrical measurements, the platinum paste was symmetrically applied to both sides of the SrZr_1−x_Lu_x_O_3−δ_ pellets and calcined at 1000 °C, 1 h. The pellets were installed on a test stand having room for three samples. The impedance measurements were performed on a BioLogic SP-200 impedance meter in the frequency range from 0.1 Hz to 1 MHz with an amplitude of 30 mV in the air with different contents of water vapor over the temperature range of 250–800 °C. Dry air (pH_2_O = 0.04 kPa) was obtained by blowing atmospheric air through a pipe filled with zeolite beads. In order to increase pH_2_O, the air flow was passed through a bubbler filled with a water, which was thermostatically controlled at 0 °C (pH_2_O = 0.61 kPa) and 24 °C (pH_2_O = 3.0 kPa).

## 3. Results and Discussion

### 3.1. Characterization of Phase Purity, Structure and Morphology 

**Figure 2:**
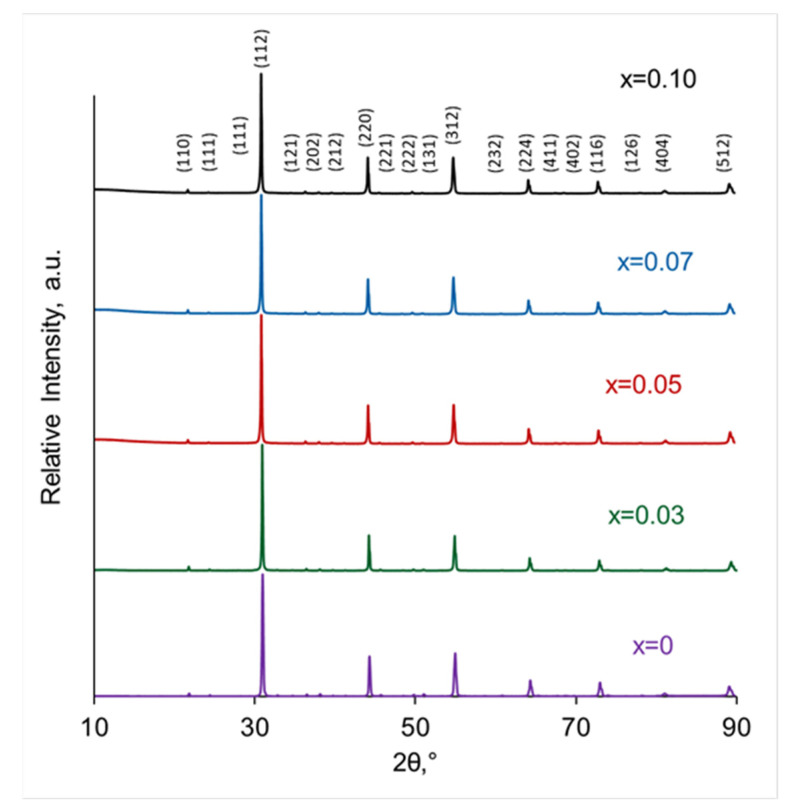
XRD patterns of SrZr_1−x_Lu_x_O_3−δ_ powders.

**Figure 3:**
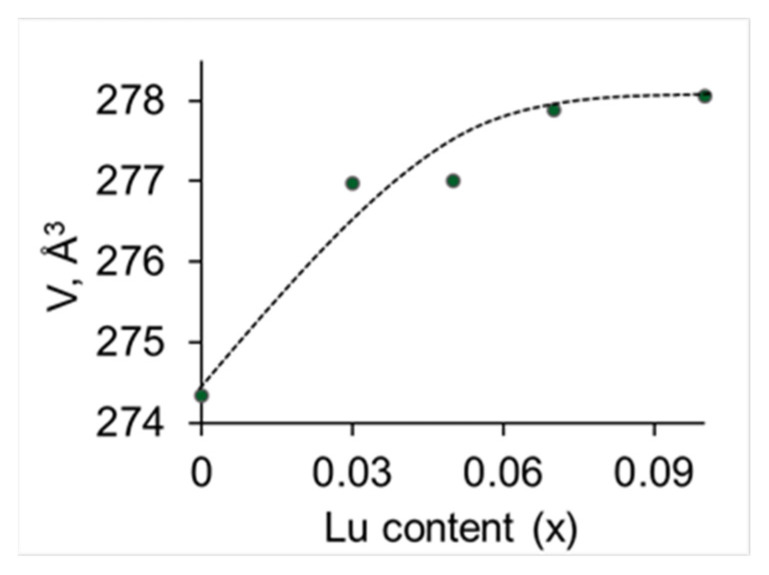
Unit cell volume of SrZr_1−x_Lu_x_O_3−δ_ vs. Lu content.

The XRD patterns of SrZr_1−x_Lu_x_O_3−δ_ powders are shown in Figure 2. All diffractograms match the reference diffraction pattern for orthorhombic SrZrO_3_ (PDF 01-0074-2231). The XRD results confirm that the synthesis was complete and no raw materials remain unreacted. The lattice parameters a, b and c were calculated using SmartLab Studio II software, and the unit cell volume was calculated as the product of these parameters. The unit cell volumes for each composition are shown in Figure 3. As can be seen, the cell volume significantly increases with the addition of a small amount of Lu (x = 0.03), which is consistent with the idea that Lu^3+^ ions with a large ionic radius (0.86 Å in 6-fold coordination) replace Zr^4+^ ions with a smaller radius (0.72 Å in 6-fold coordination), and slightly increases with a further increase in the dopant content. The weak dependence of the unit cell volume on Lu content at x > 0.03 may be due to the following reasons: (i) Lu content is beyond the solubility limit which should result in the generation of a minor phase not yet detected by X-rays; (ii) Lu^3+^ ions are distributed over the positions of two types, namely Zr-sites and Sr-sites. The substitution of Zr^4+^ with Lu^3+^ leads to an increase in the cell volume, while the substitution of Sr^2+^ ions which have a large radius (1.44 Å in 12-fold coordination) with Lu^3+^ should result in a decrease in the cell volume. Therefore, if Lu^3+^ ions substitute for both Zr-sites and Sr-sites, the size effects should partially compensate for each other. The phenomenon of dopant distribution over A- and B- sites in the perovskite ABO_3_ structure has been reported earlier [11,20,21,22,23]. It is obvious that the size of the dopant ion affects its localization in the structure; for example, it was shown by using Rietveld refinement of XRD data for the BaZr_0.8_M_0.2_O_3−δ_ (M = Sc, Eu, Sm, Dy) series that Sc ions occupied B-sites only, while larger Eu, Sm, and Dy ions incorporated on both A- and B-sites of BaZrO_3_ structure [22]. Using the density functional theory calculations for Y- and Sc-doped SrZrO_3_ perovskites, it was shown that smaller Sc ions replace Zr ions, but larger Y ions incorporate on both Zr- and Sr-sites [10]. The conductivity behavior and the Raman spectra of Sr_x_Zr_0.95_Y_0.05_O_3−δ_ ceramics indicated that Sr deficiency promoted the incorporation of Y ions on Sr-sites [11,23]. Thus, both (i) and (ii) scenarios can be realized when doping with lutetium. 

**Figure 4:**
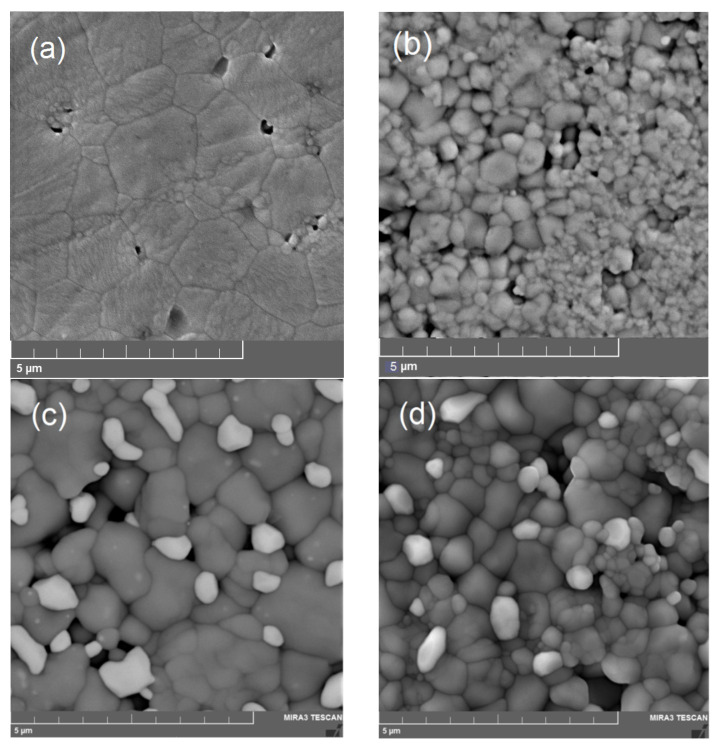
SEM images of sintered SrZr_1−x_Lu_x_O_3−δ_ samples: (**a**) x = 0, (**b**) x = 0.03, (**c**) x = 0.07, and (**d**) x = 0.10.

SEM images of the SrZr_1−x_Lu_x_O_3−δ_ surface presented in Figure 4 show the granular microstructure of the ceramics. As can be seen, the samples with x = 0 and 0.03 are homogeneous and consist of similar grey grains; while an increase in Lu content leads to the precipitation of a secondary phase, which appears in the form of light grey grains. Analysis of the elemental composition by using the EDX method revealed that the main phase has a composition close to the nominal one, and the light grey grains are composed mainly of strontium, lutetium, and oxygen. The average composition of the main and minor phases of SrZr_1−x_Lu_x_O_3−δ_ ceramics are summarized in Table 1. Analysis of the SEM and EDX data allowed us to conclude that the solubility limit of Lu in SrZr_1−x_Lu_x_O_3−δ_ lies between x = 0.03 and 0.05, which is consistent with the dependence of the unit cell volume on Lu content. However, the possibility of incorporation of the dopant ions into the positions of Sr cannot be excluded, but this task is beyond the scope of this study.

**Figure 5:**
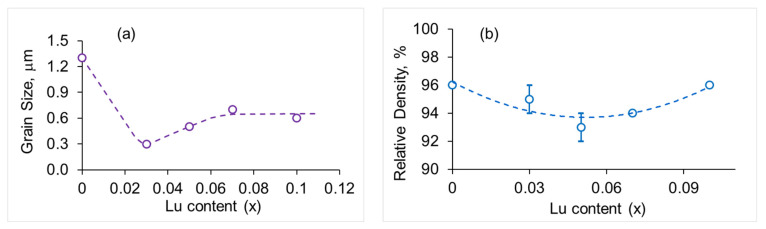
Average grain size (**a**) and relative density (**b**) of SrZr_1−x_Lu_x_O_3-_δ ceramics vs. Lu content.

Analysis of the SEM images of SrZr_1−x_Lu_x_O_3−δ_ ceramics showed that Lu-doping results in a significant decrease in the average grain size, which was evaluated by the intersection method. In the undoped sample, the average grain size is about 1.3 μm with the largest grains of ~2.5 µm. In the sample with x = 0.03, the average grain size drops to about 0.3 μm. With a further increase in Lu content, the minor phase with grains of ~0.4 µm appears, while the grains of the main phase increase to ~0.7 µm and ~0.6 µm at x = 0.07 and 0.10, respectively. The average grain size versus Lu content is shown in Figure 5a. The reasons for the decrease in grain size in the doped perovskites are not clear; however, such facts have been described in the literature (see e.g., [24]). The relative density of SrZr_1−x_Lu_x_O_3−δ_ ceramics also nonmonotonically depends on the concentration of Lu: it decreases with increasing Lu content at x < 0.05 and increases at higher values of x, as shown in Figure 5b. The revealed microstructural features can be explained by the influence of the minor Lu-rich phase, which appears at x ≥ 0.05 and enhances the grain growth and density of the ceramics. 

### 3.2. Electrical Conductivity of SrZr_1−x_Lu_x_O_3−δ_

**Figure 6:**
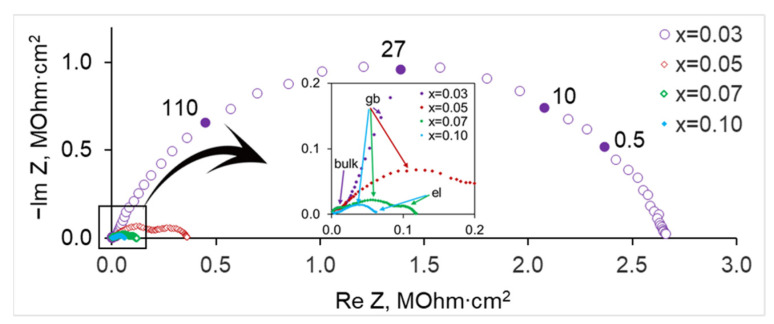
Impedance spectra of SrZr_1−x_Lu_x_O_3−δ_ ceramics in dry air (pH_2_O = 0.04 kPa) at 300 °C. Numbers near a hodograph are frequencies in Hz; in the insert: “bulk”, “gb” and “el” mark the arcs related to the grain bulk, grain boundary, and electrode polarization responses.

The electrical conductivity of SrZr_1−x_Lu_x_O_3−δ_ samples was studied in the air with different humidity values (pH_2_O = 0.04, 0.61 and 3.0 kPa) over the temperature range of 250–800 °C. The impedance spectra of the samples with different concentrations of Lu measured in dry air at 300 °C are displayed in Figure 6. At low temperatures, the Nyquist plots consist of three semicircles, and they were fitted using the equivalent circuit consisting of three parallel elements with a resistor R and a constant phase element Q, connected in series, by using ZView software. The high-frequency semicircle, which has a characteristic capacitance of ~10^–11^ F/cm^2^, can be ascribed to the contribution of grain bulk, the medium frequency arc with a capacitance of ~10^–9^ F/cm^2^ is due to the grain boundaries, and the low-frequency response with a capacitance of ~10^−7^ F/cm^2^ can be related to the electrode polarization.

**Figure 7:**
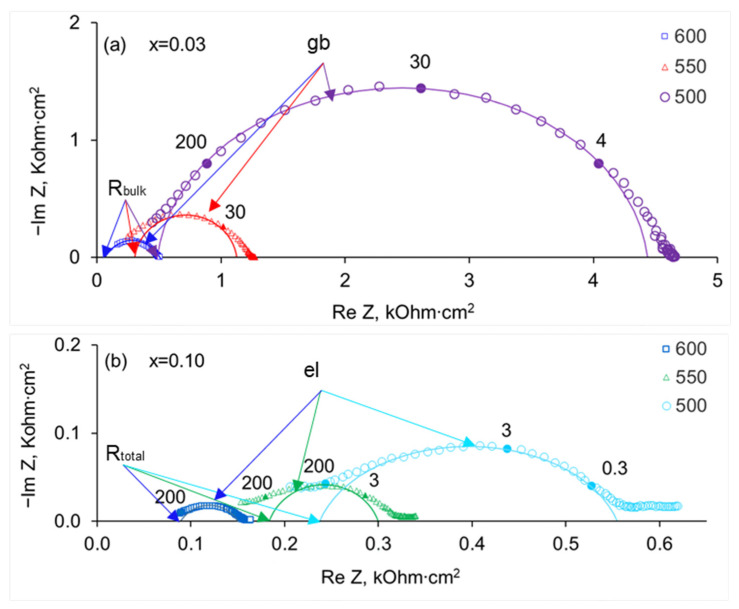
Impedance spectra of SrZr_1−x_Lu_x_O_3−δ_ ceramics at 500–600 °C in dry air (pH_2_O = 0.04 kPa): (**a**) x = 0.03, (**b**) x = 0.10. Numbers near hodographs are frequencies in kHz; R_bulk_ denotes the resistance of grain bulk, R_total_ is the total resistance of a sample, and “gb” and “el” mark the arcs related to the grain boundary and electrode polarization responses.

As can be seen in Figure 6, the resistance of the sample with x = 0.03 is the largest among the Lu-doped ceramics, and the grain boundary arc dominates so that the electrode polarization response appears just as the distortion of the low-frequency part of the grain boundary arc; at high frequencies, a small arc starting from the origin and related to the grain bulk response can be seen (see the insert in Figure 6). Figure 7 illustrates the changes in hodographs with temperature. With increasing temperature, the response of the grain bulk of the sample with x = 0.03 goes beyond the frequency range of the device, and the bulk resistance can be determined by extrapolating the high-frequency part of the semicircle related to the grain boundaries to the x-axis (see Figure 7a). For the composition with x = 0.10, only the electrode-related arc appears at high temperatures, and the total resistance of the sample can be evaluated by extrapolation (Figure 7b). 

**Figure 8:**
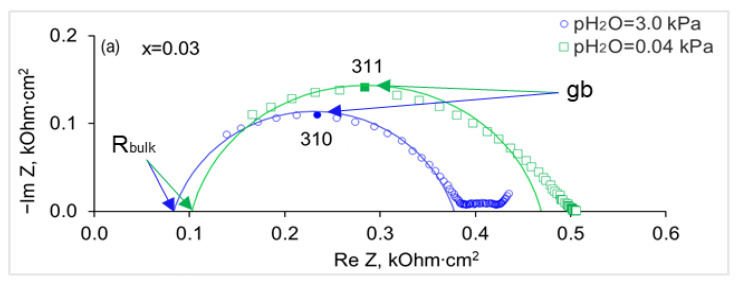

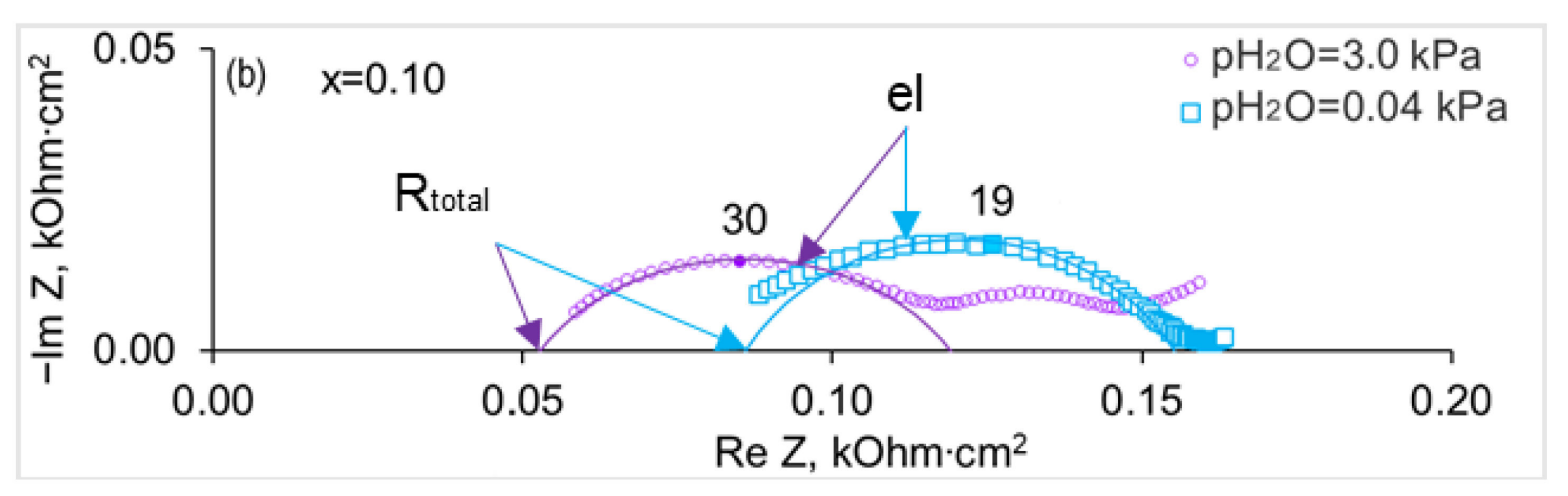
Impedance spectra of SrZr_1−x_Lu_x_O_3−δ_ ceramics at 600 °C in dry (pH_2_O = 0.04 kPa) and wet (pH_2_O = 3.0 kPa) air: (**a**) x = 0.03, (**b**) x = 0.10. Numbers near hodographs are frequencies in kHz; R_bulk_ denotes the resistance of grain bulk, R_total_ is the total resistance of a sample, and “gb” and “el” mark the arcs related to the grain boundary and electrode polarization responses.

Figure 8 demonstrates the influence of the water vapor partial pressure on the shape of hodographs. Analysis of the impedance spectra of the SrZr_1−x_Lu_x_O_3−δ_ ceramics in dry (pH_2_O = 0.04 kPa) and wet (pH_2_O = 3.0 kPa) air at 600 °C indicates that with increasing the air humidity the resistance of the samples decreases, while the polarization resistance increases. A similar behavior of the Pt electrode polarization was observed in the study of Yb-doped SrZrO_3_ [17]. The increase in the electrode polarization in wet air was supposed to be caused by a decrease in the concentration of holes in the oxide because of the hydration process. 

**Figure 9:**
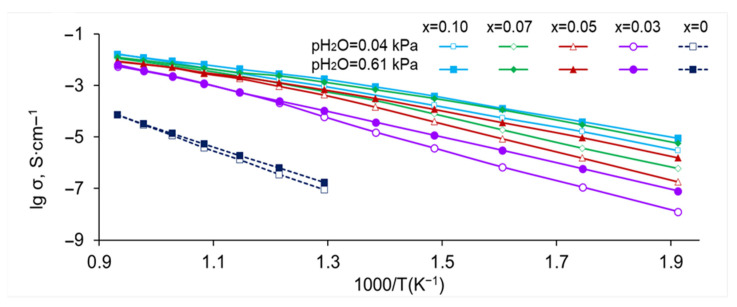
Arrhenius plots of the total conductivity of SrZr_1−x_Lu_x_O_3−δ_ in dry (pH_2_O = 0.04 kPa) and wet (pH_2_O = 0.61 kPa) air.

**Figure 10:**
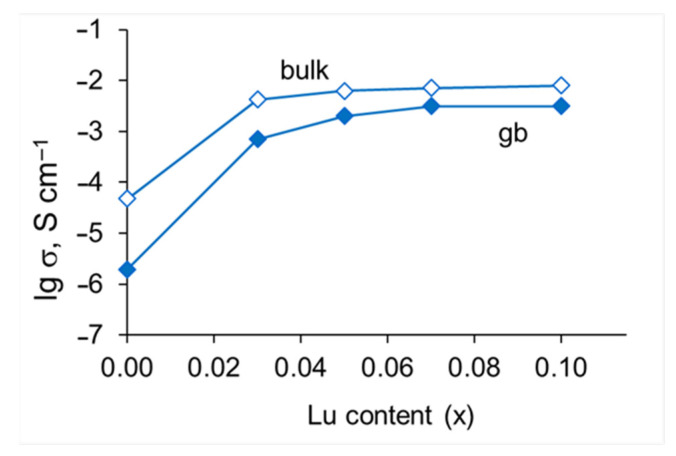
The conductivity of SrZr_1−x_Lu_x_O_3−δ_ C in wet air (pH_2_O = 3.0 kPa) at 600 °C.

The Arrhenius plots of SrZr_1−x_Lu_x_O_3−δ_ in dry and humid air are presented in Figure 9. As can be seen, the conductivity increases with pH_2_O, which is consistent with the mechanism of formation of protonic defects upon the oxide hydration (Reaction 2). Doping with a small amount of Lu (x ≤ 0.05) gives an increase in conductivity by about three orders of magnitude (at 600 °C), while the increase in Lu content to x = 0.10 enhances the conductivity only about two times. To discuss the effect of Lu content on the transport properties of grain interior and grain boundaries, the conductivities of grain bulk and grain boundaries as the functions of Lu content in wet air (pH_2_O = 3.0 kPa) at 600 °C are presented in Figure 10. As can be seen, the bulk conductivity increases by about order orders of magnitude with the addition of a small amount of Lu (x = 0.03), which is obviously caused by an increase in the oxygen vacancy concentration upon the substitution of Zr for Lu, and remains almost constant at x ≥ 0.05 which can be explained by the fact that the solubility limit of lutetium has been reached and, accordingly, the composition of the grain interior does not change with x. The grain boundary conductivity rises even more significantly with the addition of a small amount of lutetium which is probably due to the changed composition of the grain boundaries; in the range of x > 0.05, the conductivity continues to grow, albeit slightly, apparently due to an increase in the grain size and density of the ceramic. So, the conductivity behavior of SrZr_1−x_Lu_x_O_3−δ_ ceramics is consistent with the SEM, EDX, and XRD results, which indicate that the solubility limit of lutetium lies between x = 0.03 and 0.05. 

**Figure 11:**
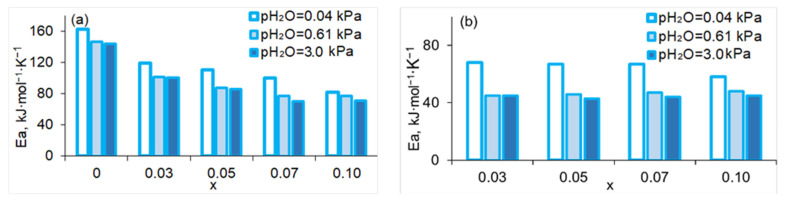
Activation energies of total (**a**) and bulk (**b**) conductivity of SrZr_1−x_Lu_x_O_3−δ_ at pH_2_O = 0.04, 0.61 and 3.0 kPa.

The activation energies of the total and bulk conductivities of SrZr_1−x_Lu_x_O_3−δ_ are summarized in Figure 11. For the undoped sample, reliable determination of grain bulk properties was difficult because of the high resistance of grain boundaries. For all compositions, the activation energy tends to decrease when the water vapor partial pressure increases, which can be explained by the increasing contribution of proton conductivity. The activation energy of the bulk conductivity just slightly varies with changing x, while that of the total conductivity decreases with increasing Lu content. The behavior of activation energy is consistent with the assumption made above about the positive effect of the Lu-containing phase, which is formed at x > 0.03, on ceramic sintering.

Analysis of the electrical conductivity data of SrZr_1−x_Lu_x_O_3−δ_ shows that the composition with x = 0.10 demonstrates the highest conductivity (10 mS cm^–1^ at pH_2_O = 0.61 kPa, 700 °C), which can be explained by the high concentration of oxygen vacancies generated by the doping and the improved microstructure, namely an increased grain size and density, due to the improved sinterability in the presence of the Lu-rich phase. 

**Figure 12:**
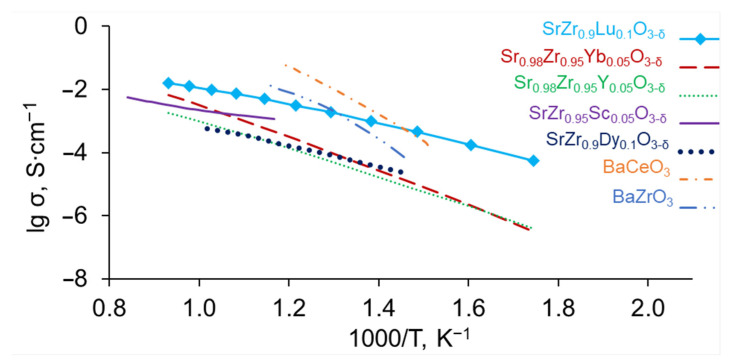
Arrhenius plots of the conductivity of SrZr_0.9_Lu_0.1_O_3−δ_ (present research), SrZr_0.95_Sc_0.05_O_3−δ_ (adapted from [25]), SrZr_0.95_Y_0.05_O_3−δ_ (adapted from [11]), SrZr_0.9_Dy_0.1_O_3−δ_ (adapted from [18]), SrZr_0.95_Yb_0.05_O_3−δ_ (adapted from [17]), BaCeO_3_ and BaZrO_3_ (adapted from [26]), in wet air.

To compare the properties of SrZr_1−x_Lu_x_O_3−δ_ with other proton-conducting membranes, Figure 12 demonstrates the conductivities of barium cerate, barium zirconate, and strontium zirconates doped with different rare earth elements. As can be seen, doping with lutetium more effectively increases the conductivity of strontium zirconate compared to Sc, Y, Dy and Yb; and the conductivity of SrZr_0.9_Lu_0.1_O_3−δ_ is comparable to that of BaCeO_3_ and BaZrO_3_. Thus, SrZr_1−x_Lu_x_O_3−δ_ ceramics can be considered promising materials of membranes for proton-conducting electrochemical devices.

## 4. Conclusions

For the first time, the effect of Lu-doping on the microstructure, phase composition, and electrical conductivity of SrZr_1−x_Lu_x_O_3−δ_ (x = 0–0.10) was investigated via X-ray diffraction, scanning electron microscopy, energy-dispersive X-ray spectroscopy, and impedance spectroscopy. Dense ceramic samples were obtained by the solution combustion synthesis and sintering at 1650 °C for 5 h. According to the X-ray diffraction data, the samples possessed an orthorhombic perovskite-type structure. SEM and EDX data indicate that the solubility limit of Lu in SrZr_1−x_Lu_x_O_3−δ_ lies between x = 0.03 and 0.05. The average grain size of SrZr_1−x_Lu_x_O_3−δ_ ceramics drops from about 1.3 µm in the undoped composition to about 0.3 µm at x = 0.03; with a further increase in Lu content, a minor Lu-rich phase appears which promotes the grain growth to ~0.7 µm and ~0.6 µm at x = 0.07 and 0.10, respectively.

The electrical conductivity of SrZr_1−x_Lu_x_O_3−δ_ increases with Lu content so that SrZr_0.9_Lu_0.1_O_3−δ_ exhibits the highest conductivity (10 mS cm^–1^ at 700 °C, pH_2_O = 0.61 kPa). Doping with a small amount of Lu (x ≤ 0.05) gives an increase in conductivity by about three orders of magnitude (at 600 °C) which can be explained by the generation of oxygen vacancies, while a further increase in Lu content to x = 0.10 enhances the conductivity only about two times which is supposed to be caused mostly by the improved microstructure of the ceramic. The conductivity of SrZr_0.9_Lu_0.1_O_3−δ_ increases with the water vapor partial pressure which indicates a significant contribution of proton conductivity to charge transfer.

Doping with Lu results in a larger increase in conductivity of SrZrO_3_ compared to other rare earth elements (Sc, Y, Dy, Yb), so that the conductivity of the most conductive composition among SrZr_1−x_Lu_x_O_3−δ_ (x = 0–0.10) series, SrZr_0.9_Lu_0.1_O_3−δ_, is comparable to those of BaCeO_3_ and BaZrO_3_. Considering the high chemical stability of zirconates, SrZr_0.9_Lu_0.1_O_3−δ_ ceramics is the promising material of membranes for proton-conducting electrochemical devices.

## Figures and Tables

**Table 1:** Chemical composition of SrZr_1−x_Lu_x_O_3−δ_ ceramics.

Nominal Composition	Composition of Main Phase	Minor Phase
SrZrO_3_	Sr_0.99_Zr_1.00_O_3−δ_	−
SrZr_0.97_Lu_0.03_O_3−δ_	Sr_1.00_Zr_0.96_Lu_0.03_O_3−δ_	−
SrZr_0.95_Lu_0.05_O_3−δ_	Sr_1.00_Zr_0.96_Lu_0.04_O_3−δ_	(Sr, Lu)O_y_
SrZr_0.93_Lu_0.07_O_3−δ_	Sr_1.00_Zr_0.93_Lu_0.04_O_3−δ_	(Sr, Lu)O_y_
SrZr_0.90_Lu_0.10_O_3−δ_	Sr_1.00_Zr_0.94_Lu_0.04_O_3−δ_	(Sr, Lu)O_y_

## Data Availability

Not applicable.
